# Terrestrial mammalian wildlife responses to Unmanned Aerial Systems approaches

**DOI:** 10.1038/s41598-019-38610-x

**Published:** 2019-02-14

**Authors:** Emily Bennitt, Hattie L. A. Bartlam-Brooks, Tatjana Y. Hubel, Alan M. Wilson

**Affiliations:** 1 Okavango Research Institute, University of Botswana, Maun, Botswana; 2 Structure and Motion Laboratory, Royal Veterinary College, University of London, London, United Kingdom

## Abstract

Unmanned Aerial Systems (UAS) are increasingly being used recreationally, commercially and for wildlife research, but very few studies have quantified terrestrial mammalian reactions to UAS approaches. We used two Vertical Take-off and Landing (VTOL) UAS to approach seven herbivore species in the Moremi Game Reserve, Botswana, after securing the relevant permissions. We recorded responses to 103 vertical and 120 horizontal approaches, the latter from three altitudes above ground level (AGL). We ran mixed logistic regressions to identify factors triggering (i) any response and (ii) an evasive response. We included effects of activity, altitude, direction of approach, distance, habitat, herd type, herd size, other species, target species, time, VTOL type and wind strength. Response triggers were linked to altitude, distance, habitat and target species. Elephant (*Loxodonta africana*), giraffe (*Giraffa camelopardalis*), wildebeest (*Connochaetes taurinus*) and zebra (*Equus quagga*) were most affected by VTOL approach, impala (*Aepyceros melampus*) and lechwe (*Kobus leche*) were least responsive, and tsessebe (*Damaliscus lunatus*) displayed intermediate sensitivity. VTOLs flown lower than 60 m AGL and closer than 100 m horizontal distance from target animals triggered behavioural responses in most species. Enforced regulations on recreational UAS use in wildlife areas are necessary to minimise disturbance to terrestrial mammals.

## Introduction

Unmanned Aerial Systems (UAS) production is projected to be one of the most rapidly growing industries for the next decade^1^, with a large variety of fixed-wing and rotary-bladed UAS readily available for purchase. Fixed-wing UAS can fly relatively far and are generally quiet, but require space and sometimes equipment to be launched and landed^2,3^. Rotary-bladed or Vertical Take-off and Landing UAS (VTOL) can be launched and landed anywhere, hover steadily, and fly in any direction in rapid response to commands from the controller, but their battery life is lower and they are louder than fixed-wing UAS^4,5^. Most UAS come equipped with cameras or have the capacity for cameras such as GoPros (San Mateo, CA, USA) to be mounted onto a gimbal that can point the camera in any direction. The versatile flying, relatively low cost and potential for unusual, high resolution aerial photographs and footage render VTOLs very attractive to the general public^6^, particularly recreational tourists^1^, who upload footage of wildlife to internet sites. The adverse reactions of animals shown in some of these videos has resulted in concern being expressed in a number of sectors that recreational UAS use in wilderness areas could have a negative impact on wildlife^7,8^. The rapid advent of accessible UAS technology has outpaced government regulations in many countries^9^, which have either ignored UAS use or banned it completely for commercial purposes^1^, and regulations for private users remain largely undeveloped. Most countries require UAS to remain within sight of the operator and enforce restrictions on altitude to prevent interference with aircraft. Generally, UAS are additionally prohibited from approaching airfields, people and buildings. Regulations governing UAS use in wildlife areas are being implemented in a number of countries, including Botswana, South Africa and Tanzania, but objective measurements to inform safe use guidelines are sparse, so there is little information available for permitting officials to assess potential impacts of proposed projects.

UAS provide substantial benefits to research and conservation: they can be used to track radiotagged animals^10^, conduct surveys^11^ in remote areas^12^, detect cryptic individuals using thermal imaging^13^, collect locomotion data from free-ranging animals^14^, count groups using automated software^15^, estimate body mass^16^, determine gender^17^ and assist with anti-poaching efforts^18^. Many of these activities are habitually undertaken using manned aircraft, and UAS offer benefits in terms of safety^19^, noise^20^, precision^21^, response time^10^ and cost^22^. However, regulations governing UAS flight paths can be prohibitive^23^, the trade-off between battery time and payload can restrict flight time^7^, and the time required to process images from UAS flights is substantial^24^, leading to the ongoing development of automated detection and tracking software^25^.

Wildlife can respond to auditory and visual cues from UAS in negative ways^8^, and several studies have attempted to quantify thresholds for negative responses^5,26^, although many have not considered this aspect of UAS use^6,9^. Notably, Vas *et al*.^6^ conducted the first study to test the behavioural reactions of three bird species to experimental VTOL approaches under different conditions, and showed that in 80% of cases, birds were not disturbed by VTOLs flying within 4 m of them. However, most published experiments used fixed wing UAS and were conducted with marine wildlife, primarily birds^8^. Some bird species showed no disturbance, which could be linked to high ambient noise levels from the rest of the colony or from strong winds^27^. Other bird species flushed and left their nests, a reaction reminiscent of escape from aerial attack and that occasionally resulted in egg predation^22^. Most marine mammals showed very little response to UAS approaches, the sound of which were attenuated by water^2^ and often below their hearing threshold, but pinnipeds, which spent some of their time on land, were more sensitive than cetaceans^20^. Response strength varies with a large range of factors, including species^22^, group size^8^, behaviour^8^, breeding status^28^, and wind levels^27^.

Very few studies have looked at the impact of UAS on terrestrial mammals, so their responses are largely unknown^29^. Some data from observational rather than experimental studies suggest that they are tolerant of UAS approaches^7,8^; however other studies suggest the opposite. Black bears (*Ursus americanus*), including one hibernating individual, experienced raised heart rates in response to overhead flights by VTOLs^29^, and UAS are used to chase elephants from fields to prevent crop-raiding^30^. The sound made by VTOLs is similar to that of a bee swarm and causes elephants to move away rapidly^31^. There is therefore an urgent need for experiment-based data to quantify behavioural responses of terrestrial mammals to VTOL approaches, the UAS type most commonly used recreationally^3,7^. To quantify the disturbance levels of flying VTOLs near terrestrial mammals, we conducted experiments on seven common herbivore species in the Moremi Game Reserve, Botswana, with two commonly used quadcopter VTOL models, a Phantom III and an Inspire I (DJI, Shenzhen, China). We hypothesised that wildlife responses to VTOL approach would (i) vary with species, (ii) increase with VTOL proximity, and (iii) be stronger for larger VTOLs, in still conditions, for larger group sizes, in open habitat, and when animals were active.

## Results

We recorded response data from 103 vertical and 120 horizontal approaches, including 45, 34 and 42 approaches at 10, 20 and 30 m AGL, respectively (Table 1). Mean launch distance ± S.D. from target animals was 301 ± 190 m. 97.1% and 94.2% of vertical and horizontal approaches, respectively, were to herds of ≥2 animals. Two levels of response were recorded from some approaches, when animals responded with vigilance followed by active avoidance. 41.7% and 9.2% of vertical and horizontal approaches were preceded by horizontal and vertical approaches, respectively. Vertical approaches included hovering directly above target animals for a mean ± S.D of 50.8 ± 16.0 seconds. Horizontal approaches lasted a mean ± S.D of 32 .0 ± 14.9 seconds, which included flying from the vehicle to the target animals, but not return flights after experiments. To minimise disturbance to target groups, all experiments were stopped when animals moved away from the VTOL, which was flown straight back to the origin. Total flight time was not recorded.

**Table 1:** Number of vertical and horizontal approaches by Unmanned Aerial Systems towards seven herbivore species in the Okavango Delta, Botswana. Horizontal approaches were conducted at three different altitudes.

Species	Vertical	Horizontal		
		10 m	20 m	30 m
Elephant	14	6	4	5
Giraffe	17	6	6	5
Impala	16	6	5	7
Lechwe	12	8	5	5
Tsessebe	14	6	5	5
Wildebeest	12	5	4	8
Zebra	18	7	5	7

### Vertical approach

Some target groups responded to the VTOL before it reached its starting point directly above them, so horizontal distance to the target group was recorded as well as altitude.

### No response vs. response

Model averaging of four candidate models (Supplementary Table S1) identified altitude, distance and species as the main factors determining whether target groups would respond to VTOL approaches (Table 2). A response was more likely when VTOLs were lower but further away. Confidence intervals showed that elephant, giraffe, wildebeest and zebra were more likely to respond than impala, whereas lechwe and tsessebe had similar response patterns to impala (Table 2).

**Table 2:** Model averaged parameter values explaining herbivore response levels to vertical approaches by Unmanned Aerial Systems.

Reference	Response	Parameter	Estimate	Unconditional standard error	Confidence intervals	Relative importance
No response	Response	***Altitude***	***−3***.***45***	***0***.***49***	***−4***.***42***, ***−2***.***48***	***1***.***00***
		***Distance***	***0***.***49***	***0***.***21***	***0***.***08***, ***0***.***90***	***1***.***00***
		Habitat	−0.05	0.25	−1.52, 0.86	0.14
		Size	0.20	0.28	−0.13, 0.95	0.49
		***Species_Elephant***	***3***.***57***	***1***.***21***	***1***.***19***, ***5***.***95***	***1***.***00***
		***Species_Giraffe***	***3***.***59***	***1***.***24***	***1***.***16***, ***6***.***02***	***1***.***00***
		Species_Lechwe	0.07	1.24	−2.37, 2.50	1.00
		Species_Tsessebe	2.28	1.20	−0.06, 4.63	1.00
		***Species_Wildebeest***	***3***.***48***	***1***.***23***	***1***.***08***, ***5***.***88***	***1***.***00***
		***Species_Zebra***	***3***.***21***	***1***.***21***	***0***.***83***, ***5***.***58***	***1***.***00***
		Time of day_AM	0.06	0.26	−0.71, 1.40	0.17
		Time of day_PM	−0.11	0.31	−1.61, 0.39	0.17
Vigilant	Active	Activity_Stand	0.05	0.30	−1.17, 2.53	0.07
		Activity_Drink	0.11	0.54	−1.18, 4.29	0.07
		Activity_Feed	0.08	0.37	−0.65, 2.87	0.07
		Activity_Walk	−0.01	0.26	−1.91, 1.89	0.07
		***Altitude***	***0***.***58***	***0***.***23***	***0***.***12***, ***1***.***03***	***1***.***00***
		Direction_Front	0.21	0.62	−0.85, 3.10	0.19
		Direction_Side	−0.01	0.36	−1.66, 1.62	0.19
		Direction_Behind	−0.29	0.70	−3.19, 0.12	0.19
		Distance	0.37	0.23	0.01, 0.82	0.90
		Habitat	−0.03	0.18	−1.38, 0.58	0.08
		Species_Elephant	−0.28	0.63	−1.51, 0.96	1.00
		Species_Giraffe	−0.83	0.73	−2.26, 0.59	1.00
		Species_Lechwe	−0.09	0.63	−1.35, 1.16	1.00
		Species_Tsessebe	−1.16	0.69	−2.51, 0.20	1.00
		Species_Wildebeest	−1.54	0.79	−3.09, 0.02	1.00
		***Species_Zebra***	***−3***.***51***	***1***.***18***	***−5***.***83***,***−1***.***19***	***1***.***00***
		Time of day_AM	0.14	0.33	−0.52, 1.21	0.40
		Time of day_PM	−0.32	0.51	−1.82, 0.25	0.40
		UAS type	0.09	0.26	−0.36, 1.24	0.21

Parameters with high relative importance and confidence intervals >1 or <1 are in bold italics.

### Vigilance vs. avoidance

Model averaging of nine candidate models (Supplementary Table S1) identified altitude and species as the main factors determining whether target groups would avoid a VTOL approach rather than display vigilance (Table 2). Avoidance behaviour was more likely when VTOLs were at higher altitudes. Confidence intervals showed that most species had similar response patterns to impala, but zebra were more likely to be vigilant and less likely to move than impala (Table 2).

### Response thresholds

**Figure 1:**
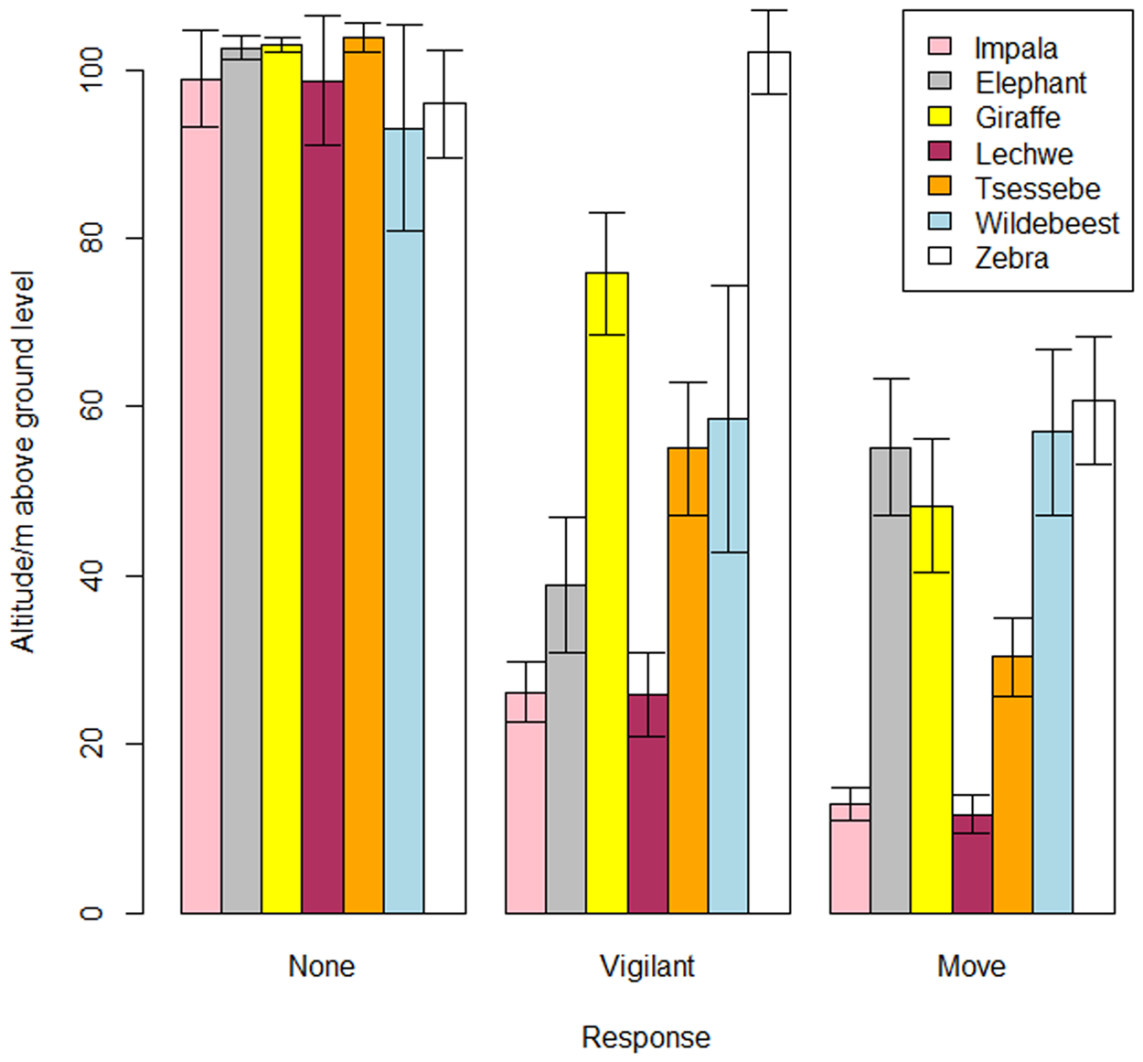
Responses to vertical Unmanned Aerial Systems approaches according to altitude above ground level by seven species of African herbivore in the Moremi Game Reserve, Botswana. Error bars represent S.E.

Vigilance thresholds in relation to altitude varied substantially with species (Fig. 1). Zebra were the most sensitive species to VTOL approach, becoming vigilant when VTOLs were >100 m AGL; giraffe, tsessebe and wildebeest became vigilant when VTOLs dropped to 50–80 m AGL; and impala, elephant and lechwe only became vigilant when VTOLs dropped to 30–50 m AGL (Fig. 1). Elephant, giraffe, wildebeest and zebra all moved to avoid VTOLs when they were approximately 50–60 m AGL, whereas tsessebe moved away when VTOLs dropped to 30 m AGL, and impala and lechwe were highly tolerant of VTOL approach, only moving when VTOLs were approximately 15 m AGL (Fig. 1). Across species, animals responded when VTOLs were at a mean ± S.D. of 42.9 ± 33.6 m AGL, but most species responded when VTOLs flew below 60 m AGL (Fig. 1).

### Horizontal approach

#### No response vs. response

Model averaging of seventeen candidate models (Supplementary Table S1) identified altitude, distance and species as the main factors determining whether target groups would respond to VTOL approaches (Table 3). A response was more likely when VTOLs were lower and closer. Confidence intervals showed that giraffe, tsessebe, wildebeest and zebra were more likely to respond than impala, whereas elephant and lechwe had similar response patterns to impala (Table 3).

**Table 3:** Model averaged parameter values explaining herbivore response levels to horizontal approaches by Unmanned Aerial Systems.

Reference	Reaction	Parameter	Estimate	Unconditional standard error	Confidence intervals	Relative importance
No response	Response	Activity_Stand	0.04	0.22	−0.36, 2.18	0.04
		Activity_Drink	−0.09	0.50	−4.77, 0.40	0.04
		Activity_Feed	0.04	0.24	−0.25, 2.27	0.04
		Activity_Walk	0.02	0.09	−0.77, 1.80	0.04
		***Altitude***	***−0***.***62***	***0***.***19***	***−0***.***95***,***−0***.***22***	***1***.***00***
		***Distance***	***−3***.***83***	***0***.***47***	***−4***.***68***,***−2***.***88***	***1***.***00***
		Habitat	−0.30	0.51	−1.79, 0.39	0. 40
		Previous exposure	−0.41	0.60	−2.07, 0.31	0.47
		Size	0.09	0.18	−0.17, 0.70	0.35
		Species_Elephant	1.41	0.77	−0.02, 3.03	1.00
		***Species_Giraffe***	***1***.***78***	***0***.***71***	***0***.***38***, ***3***.***11***	***1***.***00***
		Species_Lechwe	1.10	0.65	−0.21, 2.32	1.00
		***Species_Tsessebe***	***1***.***39***	***0***.***64***	***0***.***13***, ***2***.***65***	***1***.***00***
		***Species_Wildebeest***	***3***.***96***	***0***.***81***	***2***.***36***, ***5***.***54***	***1***.***00***
		***Species_Zebra***	***2***.***35***	***0***.***65***	***1***.***09***, ***3***.***65***	***1***.***00***
		Time of day_AM	0.18	0.37	−0.51, 1.31	0.39
		Time of day_PM	−0.19	0.37	−1.37, 0.41	0.39
		UAS type	−0.01	0.09	−1.03, 0.53	0.04
Vigilant	Active	***Distance***	***1***.***42***	***0***.***28***	***0***.***86***, ***1***.***97***	***1***.***00***
		***Habitat***	***1***.***79***	***0***.***78***	***0***.***24***, ***3***.***31***	***1***.***00***
		Herd type	−0.23	0.36	−2.16, 0.44	0.27
		Previous exposure	0.12	0.36	−0.59, 1.84	0.19
		***Species_Elephant***	***2***.***48***	***0***.***99***	***0***.***52***, ***4***.***39***	***1***.***00***
		***Species_Giraffe***	***2***.***00***	***0***.***94***	***0***.***15***, ***3***.***86***	***1***.***00***
		Species_Lechwe	0.60	0.79	−0.97, 2.15	1.00
		Species_Tsessebe	0.24	0.79	−1.31, 1.81	1.00
		Species_Wildebeest	−1.81	0.91	−3.60, 0.01	1.00
		Species_Zebra	−0.20	0. 78	−1.75, 1.34	1.00
		UAS type	0.06	0.22	−0.49, 1.21	0.17

Parameters with high relative importance and confidence intervals >1 or <1 are in bold italics.

#### Vigilance vs. Avoidance

Model averaging of four candidate models (Supplementary Table S1) identified distance, habitat and species as the main factors determining whether target groups would avoid a VTOL approach rather than display vigilance (Table 3). Avoidance behaviour was more likely when VTOLs were further away and when target animals were in open habitat. Confidence intervals showed that elephant and giraffe were more likely than impala to avoid an approach, whereas the other species had similar response patterns to impala (Table 3). Model-averaged parameters indicated that previous exposure to vertical UAS approaches may have had an effect on responses for subsequent horizontal approaches (Table 3). However, the relative importance of previous exposure was small, indicating that the variable did not play a substantial role in determining wildlife responses.

#### Response thresholds

**Figure 2:**
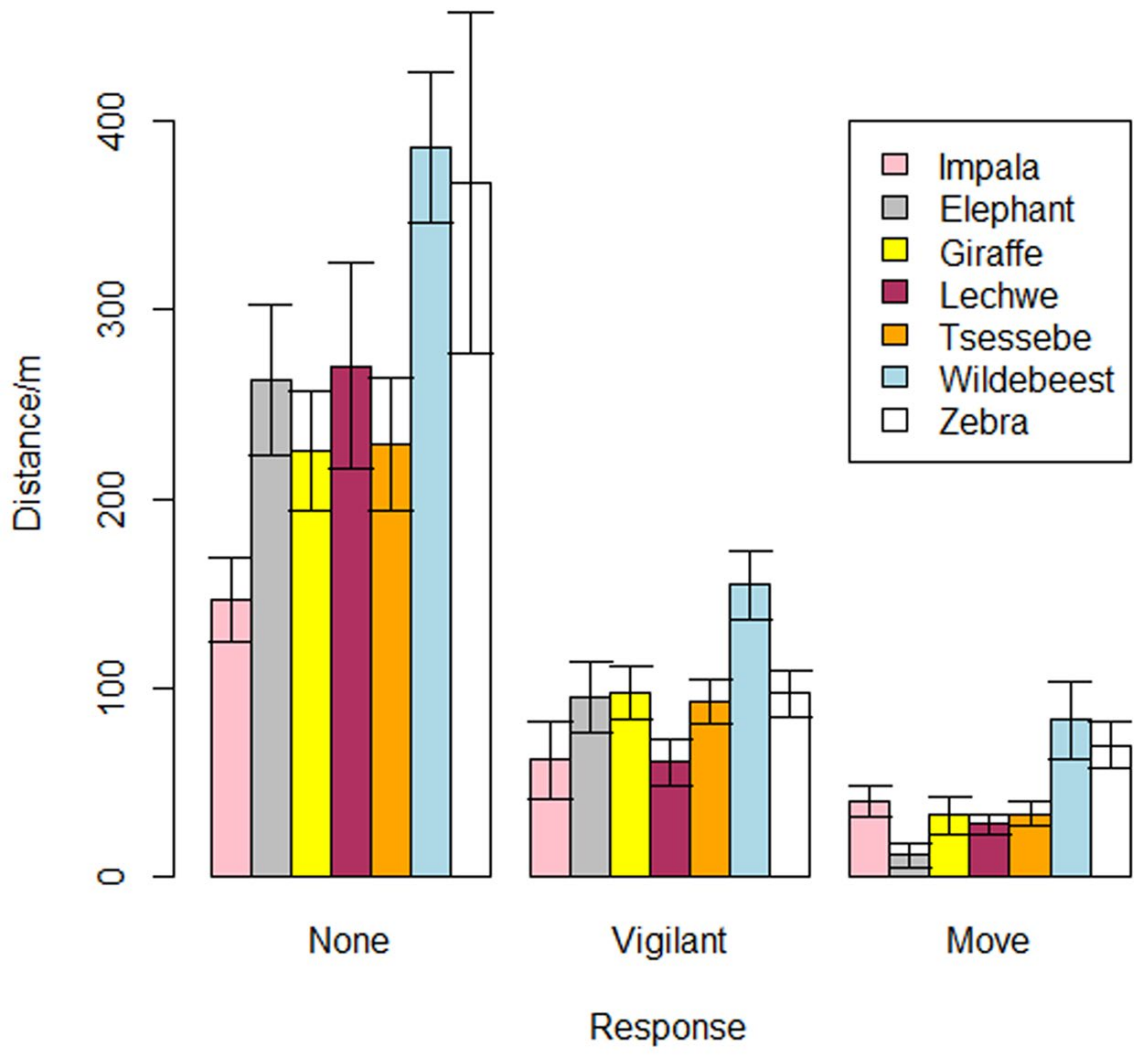
Responses to horizontal Unmanned Aerial Systems approaches according to distance by seven species of African herbivore in the Moremi Game Reserve, Botswana. Error bars represent S.E.

Vigilance thresholds in relation to distance were relatively similar for elephant, giraffe, tsessebe and zebra, which became vigilant when VTOLs were approximately 100 m away (Fig. 2). Wildebeest were the most sensitive species to VTOL approach, becoming vigilant when VTOLs were approximately 150 m away, and impala and lechwe were the most tolerant species, only becoming vigilant when VTOLs were within approximately 60 m (Fig. 2). Wildebeest and zebra moved away from VTOLs when they were at approximately 80–100 m away, whereas impala, giraffe, lechwe and tsessebe moved away when VTOLs were approximately 40–50 m away, and elephant were only likely to move away when VTOLs came within approximately 20 m of a target group (Fig. 2). Across species, animals responded when VTOLs were at a mean ± S.D. of 66.8 ± 60.0 m horizontal distance from target groups, but most species responded when VTOLs flew closer than 100 m from target groups (Fig. 2).

## Discussion

Increasing recreational UAS use has led to a need for precise UAS regulations through simple rules that are easily enforceable, including guidelines for flying UAS around wildlife^29^. Terrestrial mammals were thought to be tolerant of UAS approaches^3^ or susceptible to habituation^7^. Our results, based on dedicated experiments, showed that all study species responded to VTOL approaches negatively, although they varied in their level of response and their tolerance for VTOL proximity. Most of the environmental and situational factors predicted to affect species responses to VTOL approach had limited impact, although animals were more likely to avoid approaches than be vigilant in open habitat. Several other factors were included in model-averaged parameters but, based on confidence intervals and measures of relative importance, none had substantial effects on response levels. Previous exposure to vertical VTOL approaches had a small effect on responses to horizontal approaches, but the effects of repeated or prolonged exposure over a long time scale were not of primary interest in this short-term study. Deviance values indicated that models predicting any response were a better fit to the data than those differentiating between vigilant and active responses. Our results therefore suggest that the primary factors causing wildlife responses are vertical and horizontal VTOL proximity, regardless of VTOL type, wind levels, group size or type, or activity patterns, but response strength varies substantially with species. Our experimental design did not consider disturbance duration following VTOL approach, but this is also likely to vary with species. Our experiments were not conducted during the hottest time of year or during the breeding season, so VTOL approaches during those times could have more detrimental consequences for targeted wildlife.

Previous studies found that wildlife responded most strongly to vertical VTOL approaches, but most of these were conducted on birds under threat of aerial predation^6^. Large herbivores experience very little aerial predation because of their size, although newborns may be vulnerable to large raptors. However, the sound made by VTOLs is highly reminiscent of swarming bees, which can chase and sting wildlife of all sizes and are actively used as a deterrent for species such as elephant^32^. Our study species responded to VTOL approaches at distances large enough for VTOLs to be difficult to detect visually, so responses are likely to have been triggered by auditory rather than visual cues. However, video footage showed some giraffe becoming vigilant until the VTOL descended into their field of view, and only moving away after looking directly at the VTOL. Noise levels would have increased during descents, but animals may have had difficulty in identifying the origin of the sound, and therefore remained indecisive about the optimal direction in which to move. Target species were more likely to move than be vigilant in open habitats, whereas vigilance was preferred in wooded habitats. Prey species hunted by ambush predators experience higher predation risk in wooded habitats^33^, where sudden movements can alert predators to their presence^34^. Wooded habitats also contain more obstacles that can hinder and possibly hurt running animals^35^, so vigilance rather than avoidance may be an adaptive response to potential threats in wooded habitats.

Species varied substantially in their response levels. Elephant, giraffe, wildebeest and zebra appeared to be more sensitive to VTOL approaches than impala and lechwe, whereas tsessebe showed intermediate sensitivity. Additionally, elephant and giraffe appeared to be more sensitive to vertical approaches than horizontal ones. Impala are hunted by all predator species^36^, so should be alert for danger, but they were among the least responsive of the seven species, as were lechwe. Species vary in their hearing ability^37^, so impala and lechwe may have less sensitive hearing than other species at the sound frequency of the VTOLs, rendering them less responsive to auditory cues. The two lion groups approached with VTOLs showed aggression responses, followed by running, indicating that predators may also respond negatively to VTOL proximity. Our results highlight the need for any project using UAS to evaluate study species’ sensitivity to UAS approaches prior to beginning a study and adjust their approach characteristics accordingly^5^.

Our experiments were designed to simulate recreational VTOL approaches by operators who may wish to fly UAS as close to an animal as possible, so our horizontal approach altitudes were relatively low. The combination of vertical and horizontal approaches allowed us to identify altitude and distance thresholds that should be respected to avoid causing disturbance to wildlife, although our experimental design did not allow us to test the potential for habituation to VTOL approaches, which may be possible over time. Most of our study species responded negatively to VTOLs flying below 60 m AGL and closer than 100 m horizontal distance, consistent with identified noise detection thresholds for wildlife^8^. The high response levels of zebra and wildebeest should discourage any use of VTOLs around them. VTOLs being used to study particular species could inadvertently affect other, more sensitive species^20^, so all species in the proposed flight path must be considered before launching. Our study species were all large herbivores, but the VTOLs could also have disturbed smaller, more cryptic species that were not targeted, such as rodents or birds. We have recorded obvious responses, but there may well be less apparent responses as well, such as raised heart rates and stress levels^29^. The low level of understanding of the full range of responses by all species that could be affected by UAS flight emphasises the need for caution during their use to avoid inadvertent and potentially undetected impacts, including on non-target species. Recreational UAS users with limited knowledge of wildlife ecology are more likely to cause disturbances than trained researchers and film-makers with a thorough understanding of animal behaviour.

Wildlife responses to external stimuli, including anthropogenic disturbances such as UAS approaches, vary substantially with a wide range of variables^38^. Although we attempted to include a large variety of factors, some that could not be considered may have influenced wildlife responses. No predators were observed during experiments, but target groups could have experienced predation prior to experiments, particularly before morning sessions. Physiological factors, such as hydration levels, and hormone and stress levels were not quantifiable but could have affected wildlife responses. Experiments conducted during this study were designed to elicit responses but we did not attempt to quantify potential attenuation or accentuation of responses with repeated or prolonged exposure. Response thresholds are likely to vary according to a large array of internal and external factors, so any study that involves the use of UAS must thoroughly test their protocols under a range of conditions prior to inception.

Our results demonstrate the sensitivity of terrestrial wildlife to UAS approaches and highlight the importance of enforceable guidelines for UAS use in protected wildlife areas, guidelines that could extend to banning recreational UAS use in such areas^8^. Most tourists are not aware of the sensitivity of wildlife to UAS approaches or the negative impacts of disturbing wildlife and causing them to flee. Animals run frequently during their daily activities because of intra- and inter-specific interactions^39^. However, running from a UAS could increase the risk of predation or accidental injury and young dependent on their mothers could get left behind or trampled^22^, putting target animals’ lives at risk^29^. As UAS use increases and becomes more commonplace^1^, increased enforcement of regulations by relevant authorities may be beneficial, possibly accompanied by checks for UAS at entry points into protected areas.

However, UAS can be highly beneficial tools for conservation^7,25^, so a blanket ban of their use could prove detrimental. UAS enable the use of non-invasive techniques for monitoring and surveying wildlife, particularly in remote areas that are difficult to access^12^, and they can have many applications for research and conservation^3,7^. UAS also provide high quality aerial footage at a fraction of the cost of manned aircraft such as helicopters^21^, and are therefore an excellent tool for film-makers. Both research and film-making professionals use UAS to record natural behaviour from wildlife and therefore have different motivations to recreational UAS operators. Legal agreements between government and researchers or film-makers, with regular checks of field operations and footage, could be put in place to ensure that regulations are being followed. Recreational UAS users are unlikely to consider species-level differences in responses and could therefore have substantial impacts on wildlife, indicating that a ban on recreational UAS use in protected areas would be beneficial for wildlife. Further research is required to quantify responses of other wildlife species to VTOL and fixed-wing UAS approaches to identify any long-term effects of disturbance or habituation linked to UAS, and to develop regulations based on data from purposive studies^8^.

## Methods

### Study area

**Figure 3:**
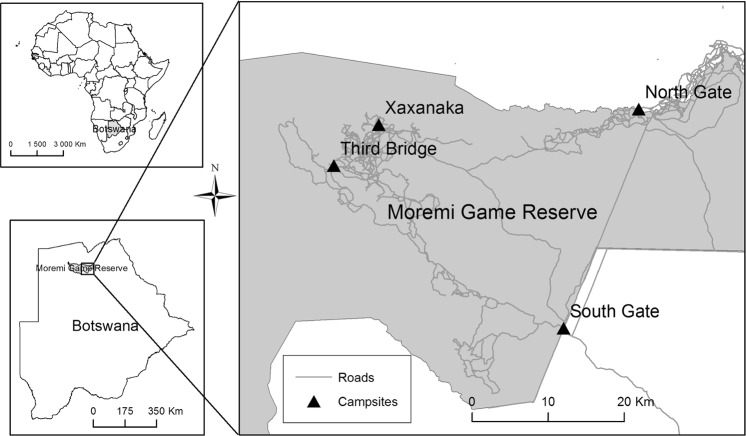
Map of study area, showing road networks and campsites in the Eastern Moremi Game Reserve, Botswana.

The study was undertaken in the Moremi Game Reserve, a protected area in northern Botswana with relatively high densities of wildlife in a mosaic of habitat types^40^. We conducted our study in the eastern Moremi Game Reserve (EMGR), which is accessible by self-driving tourists and mobile safari operators^41^ who are self-regulatory while driving along roads between campsites manned by Department of Wildlife and National Parks (DWNP) staff (Fig. 3).

In Botswana, UAS operators require annually renewable certificates issued by the Civil Aviation Authority of Botswana following a full background check and an interview with the Department of Intelligence Services. UAS use in protected areas is restricted to film makers and researchers with special dispensations. Despite existing regulations, tourists in the EMGR are regularly observed flying VTOLs near wildlife (pers. obs.), presumably to capture aerial images and footage. Videos of animals running in response to UAS approaches in the EMGR have been publicly posted on the internet.

### Study species

We selected seven commonly-occurring herbivore species of varying body size: African elephant (*Loxodonta africana*), giraffe (*Giraffa camelopardalis*), impala (*Aepyceros melampus*), red lechwe (*Kobus leche*), tsessebe (*Damliscus lunatus lunatus*), blue wildebeest (*Connochaetes taurinus*) and plains zebra (*Equus quagga*). These species were present in the study area in sufficient densities for representative sample sizes. All species occurred in mixed breeding or bachelor groups, although solitary males were sometimes observed. Some experiments on lion (*Panthera leo*) were conducted, but sample sizes were too low for analysis.

We drove along roads between South Gate and Third Bridge (Fig. 3), opportunistically locating target animal groups. The research permit allowed us to leave roads once target groups were located, so experiments were conducted out of sight of tourists to minimise disturbance. Animals in the EMGR are approached daily by vehicles, so habituation levels are relatively high. We parked at least 100 m from target groups to minimise potential disturbance from vehicular approaches. We stayed within 3 m of the vehicle during experiments to prevent animals reacting to our presence.

### Unmanned Aerial Systems

We selected VTOLs that are commonly used recreationally and commercially for aerial filming, the Phantom III Professional and the Inspire I (DJI, Shenzhen, China). Both are quadcopters with gimbal-mounted cameras that can be controlled through the DJI Go app (DJI, Shenzhen, China) on a smartphone or tablet. At 3060 g, the Inspire is substantially larger than the 1280 g Phantom and capable of greater speeds: 19 m/s horizontal and 4 m/s vertical for the Inspire, and 15 m/s horizontal and 3 m/s vertical for the Phantom (DJI, Shenzhen, China). The UAS were equipped with GPS technology and recorded video footage with time-stamped values for altitude, speed and location. The UAS operator was issued with a Remotely Piloted Aircraft certificate (RPA (A) 008) by the Civil Aviation Authority of Botswana, following compliance with all legal requirements. We were given permission to fly UAS in the MGR by the DWNP (ref WP/NAT 15/2/2 XXVII (52)).

## Experiments

Experiments were conducted between the 2^nd^–6^th^ September 2016, during the late dry season in Botswana, when vegetation cover was lowest and visibility highest^42^. Temperatures were relatively low (daytime maximum 35 °C), ensuring that flight responses by target animals would not incur heat stress. Conditions were selected to minimise potentially negative impacts of experiments designed to elicit an evasive response.

VTOLs reached experimental altitude, as recorded by the VTOLs’ sensors, before moving towards target animals. Vertical approaches were made from >100 m above ground level (AGL), directly above target animals; horizontal approaches were made at 10, 20 or 30 m AGL. VTOL launch site GPS coordinates were recorded, as were coordinates of target animals, which were obtained from VTOL flight data for vertical approaches and from a vehicular Garmin Oregon GPS (Olathe, Kansas, U.S.A.) for horizontal approaches, following identification of landscape features at the original location of target animals. We reviewed VTOL footage with flight data and recorded VTOL GPS coordinates for every response by target animals. We converted decimal degrees from the VTOL records to Universal Transverse Mercator coordinates to allow distance calculations. We used the “foreach” package in R v3.4.1 (R Core Development Team, 2017) to calculate Euclidean distance between launch site and target animals, and between target animals and VTOL location at time of response. All approaches were made at maximum speed: 15 m/s horizontal and 3 m/s vertical for the Phantom, and 19 m/s horizontal and 4 m/s vertical for the Inspire. The VTOL flew directly towards target animals until a response was observed, or until the VTOL was above the animal.

Experiments were conducted outside of the breeding season, so there were no small offspring or heavily pregnant females in the groups. Several groups experienced vertical and horizontal approaches within an hour of each other, but no groups were knowingly re-approached on the same day. Non-target groups may have experienced secondary stimuli during approaches on target groups in the same area. All experiments followed the guidelines for the use of wild mammals in research from the American Society of Mammalogists^43^ and were carried out under ethical approval from the Ethics and Welfare Committee of the Royal Veterinary College, London (URN 2013 1233).

### Variables

For every experiment, we recorded target species and time, split into morning (6:00–10:00), midday (10:00–14:00) and afternoon (14:00–18:00). We determined whether the target group was a mixed breeding group or a bachelor group, and recorded whether they were resting (lying down), standing, feeding, drinking, or walking at the time of approach. We recorded habitat type as open or wooded, with the former including grassland and floodplains, and the latter including various woodland types^44^. We recorded wind strength as none, weak, medium or strong, based on vegetation movement. We recorded any other species within the vicinity of the target species. From the video footage, we recorded group size and determined whether the approach was to the front, side or rear of the target group. We recorded whether a group had previously experienced a VTOL approach to account for possible variation in sensitivity to VTOL approaches. When reviewing the video footage, we recorded responses as None, Vigilant or Move. None was recorded when no animals within the group changed their behaviour. Experiments were designed to identify any sign of disturbance, so Vigilant was recorded when at least one animal within a group adopted a vigilant stance or ceased their original activity. Vigilant also included head shakes for elephant, a species-specific sign of irritation without movement^45^. Move was recorded when at least one animal within a group began to walk or run. No groups were approached when they were running, but some groups were already walking when approached, so Move was recorded if they increased their pace to a higher gait.

### Statistical analyses

We separated data into vertical and horizontal approaches. Next, we ran two consecutive logistic regressions on each approach type according to progressive response classification^8^. In the first model, we designated No Response as “0” and any response (Vigilant or Move) as “1” to determine which variables caused animals to respond in any way to VTOL approaches. In the second model, we only retained data associated with responses and designated Vigilant as “0” and Move as “1”, to determine which variables caused an avoidance response. Vigilance and avoidance were not mutually exclusive responses because some groups displayed vigilance prior to moving. However, other groups displayed no response at all or only vigilance or avoidance, so the behaviour pattern was not uniform. We therefore selected this analytical approach over multinomial or ordinal regression to allow the identification of thresholds associated with any response by wildlife, as well as thresholds associated with active avoidance. Logistic regressions were used because data were binary.

All models were mixed logistic regressions with group ID as the random variable to account for multiple reactions from a given group, and fixed effects of activity, altitude, direction of approach, distance, habitat, herd type, herd size, other species, target species, time of day, UAS type and wind strength. For each multi-levelled categorical variable, the reference category was the one for which target groups were least likely to have a response based on existing literature: resting for activity; front and above for horizontal and vertical approaches, respectively, for direction; midday for time of day; and none for wind. Observations during data collection identified impala as the least responsive species, so they were the reference category for species. Full models with all variables were run in R v 3.4.1 (R Core Development Team 2017) using the ‘lme4’ package^46^. The ‘dredge’ function from the ‘MuMIn’ package was used to identify all candidate models with ΔAIC < 2, and the ‘model.avg’ function from the same package was used to estimate model averaged parameters. Parameters with higher relative importance had a stronger effect on target animal responses^47^. For parameters with multiple categories, confidence intervals spanning 1 indicated that particular category did not show a different level of response to the reference category.

## Supplementary information

Supplementary Table S1

## Data Availability

Data are available from Dryad digital repository (10.5061/dryad.83m50j8).
