# Supplementary material for: Terrestrial mammalian wildlife responses to Unmanned Aerial Systems approaches
Source: Sci Rep. 2019 Feb 14;9:2142. doi: 10.1038/s41598-019-38610-x (PMC6375938; doi:10.1038/s41598-019-38610-x)
Supplement: Supplementary file 1 — Supplementary Table S1 [file 41598_2019_38610_MOESM1_ESM.pdf]

Terrestrial mammalian wildlife responses to Unmanned Aerial Systems approaches

Emily Bennitt[1]*, Hattie L.A. Bartlam-Brooks[2], Tatjana Y. Hubel[2] and Alan M. Wilson[2]

[1]Okavango Research Institute, University of Botswana, Maun, Botswana

[2]Structure and Motion Laboratory, Royal Veterinary College, University of London, United Kingdom

*Correspondence author: Emily Bennitt; Okavango Research Institute, Private Bag 285, Maun, Botswana; ebennitt@ub.ac.bw

Supplementary Table S1. Competitive (ΔAIC ≤ 2) and null models explaining wildlife responses to vertical and horizontal approaches by Unmanned Aerial Systems (UAS). AIC = Akaike's Information Criterion; ω = AIC weights; k = number of parameters.

Approach	Response	Model	ΔAIC	ω	k	Deviance
Vertical	None vs. Any	Altitude + Distance + Size + Species	0.00	0.35	5	157
		Altitude + Distance + Species	0.09	0.34	4	159
		Altitude + Distance + Species + Time of Day	1.41	0.17	5	156
		Altitude + Distance + Habitat + Size + Species	1.89	0.14	6	157
		Null model	170.50	0.00	1	347
	Vigilant vs. Avoid	Altitude + Distance + Species	0.00	0.18	4	196
		Altitude + Distance + Species + Time of Day	0.14	0.17	5	192
		Altitude +Dir+ Distance + Species + Time of Day	0.87	0.12	6	186
		Altitude + Distance + Species + Time of Day + UAS type	0.99	0.11	6	190
		Altitude + Species	1.28	0.10	3	200
		Altitude + Distance + UAS type	1.28	0.10	4	195
		Altitude + Distance + Habitat + Species	1.62	0.08	5	196
		Activity + Altitude + Distance + Species	1.95	0.07	5	189

		Altitude + Direction + Distance + Species	1.98	0.07	5	191
		Null model	3.00	0.00	1	217
Horizontal	None vs. Any	Altitude + Distance + Species	0.00	0.09	4	221
		Altitude + Distance + Previous exposure + Species + Time of Day	0.25	0.08	6	215
		Altitude + Distance + Species + Time of Day	0.40	0.08	5	217
		Altitude + Distance + Previous exposure + Species	0.47	0.07	5	219
		Altitude + Distance + Size + Species	0.57	0.07	5	219
		Altitude + Distance + Habitat + Species	0.58	0.07	5	219
		Altitude + Distance + Habitat + Previous exposure + Species + Time of Day	0.67	0.07	7	213
		Altitude + Distance + Habitat + Previous exposure + Species	0.70	0.06	6	217
		Altitude + Distance + Habitat + Herd size + Species	0.86	0.06	6	218
		Altitude + Distance + Habitat + Previous exposure + Herd size + Species	1.11	0.05	7	216
		Altitude + Distance + Previous exposure + Herd size + Species	1.19	0.05	6	218
		Altitude + Distance + Habitat + Species + Time of Day	1.20	0.05	6	216
		Altitude + Distance + Previous exposure + Herd size + Species + Time of Day	1.62	0.04	7	214
		Altitude + Distance + Herd size + Species + Time of Day	1.63	0.04	6	216

	Activity + Altitude + Distance + Species	1.64	0.04	5	214
	Altitude + Distance + Species + UAS type	1.76	0.04	5	221
	Altitude + Distance + Habitat + Previous exposure + Herd size + Species + Time of Day	1.79	0.04	8	212
	Null model	175.2	0.00	1	413
Vigilant vs. Avoid	Distance + Habitat + Species	0.00	0.37	4	169
	Distance +Herd Type+ Habitat + Species	0.59	0.27	5	167
	Distance + Habitat + Previous + Species	1.31	0.19	5	168
	Distance + Habitat + Species + UAS type	1.58	0.17	5	168
	Null model	27.00	0.00	1	212
